# Managing Employees Undergoing Total Hip and Knee Replacement: Experiences of Workplace Representatives

**DOI:** 10.1007/s10926-018-9805-7

**Published:** 2018-08-21

**Authors:** Fiona Nouri, Carol Coole, Melanie Narayanasamy, Paul Baker, Sayeed Khan, Avril Drummond

**Affiliations:** 1 School of Health Sciences, Medical School, Queens Medical Centre, University of Nottingham, Nottingham, NG7 2UH UK; 2 South Tees NHS Hospitals Trust, James Cook University Hospital, Middlesbrough, TS3 4BW UK; 3 The Buckingham Centre, Collingwood Health, 30 Bradford Road, Slough, SL1 4PG UK

**Keywords:** Employers, Return to work, Joint replacement, Employer perspective, Work, Qualitative research

## Abstract

*Introduction* There is little research on return to work (RTW) from a workplace perspective following hip and knee replacement (THR/TKR) despite employers and other workplace personnel having a key role. Our aim was to explore the experiences of individuals in the workplace in managing employees undergoing THR/TKR. *Methods* Employers and other workplace representatives from a cross-section of employment sectors and sizes, with experience of managing employees undergoing THR/TKR in the previous 12 months, were recruited. Interviewees included small business owners, line managers, colleagues, human resources managers and occupational health advisers. Semi-structured, qualitative interviews were conducted and data were analysed thematically. *Results* Twenty-five individuals were interviewed. The main themes identified were accommodating the employee, and barriers and facilitators to RTW. Accommodations included changes to the work environment, amended duties, altered hours, changed roles and colleague support. Perceived barriers and facilitators to RTW included the role of GPs and occupational health, surgical issues, characteristics of the work environment and of employees. *Conclusions* Employers are motivated to effect supported RTW for employees undergoing THR/TKR but have insufficient guidance. Strategies are required to signpost employers to existing RTW advice, and to develop recommendations specific to lower limb arthroplasty. Communication between medical practitioners and employers should be facilitated in order to enhance the RTW experience of individuals undergoing THR/TKR.

## Introduction

Joint replacement is a cost-effective and efficient method of relieving pain, and improving function and health related quality of life, for people with arthritis of the hip and knee [1]. Arthritis-related loss of physical function is associated with unemployment, reduced income and increased sickness absence [2]. In a survey investigating the impact of osteoarthritis (OA), Fautrel et al. found that OA has a substantial impact on work, with 20% of patients surveyed still in the workforce and two-thirds of those reporting that OA was affecting their work [3]. These factors, in combination with an ageing workforce and changes to the pension age, have resulted in an increase in the number of hip and knee replacements carried out on people of working age over the past 10 years. In 2015, 17,293 of 84,462 (20%) hip replacements and 16,121 of 94,437 (17%) knee replacements performed in England, Wales and Northern Ireland were in people aged under 60 years; 25,249 (30%) hip replacements and 32,321 (34%) knee replacements were performed on inpatients aged between 60 and 69 years [4]. Projections from 2005 suggest that by 2030, the demand for primary total hip (THR) and knee (TKR) replacements will increase by 174% and 673% respectively [5]. Consequently return to work (RTW) will be a priority for an increasing proportion of the population following surgery.

A recent systematic review reported that 71–98% of patients returned to work after THR/TKR with the length of absence being between 2 and 14 weeks [6]. However, it is unclear why there is such discrepancy, or whether RTW as reported in studies, was full or sustainable. Extended periods of sickness absence can result in work disability, poorer general health, increased mental health risk and increased mortality [7]. The costs are borne by the employee, employer and society as a whole due to loss of productivity, use of health care resources and statutory financial compensation. Early, sustainable RTW should therefore have potential health and socioeconomic benefits. However, the interplay between employees, employers and surgical intervention is complex, with RTW being influenced by a range of factors [6]. Active workplace involvement is essential to successful RTW with managers playing the key role in initiating effective support strategies [8–10]. Guidance on the management of sickness absence has been published by the Department of Work and Pensions [11] recommending early employer intervention, and that employees can RTW before they are completely recovered if simple adjustments are made. However, interview studies with employers suggest that they have difficulty performing this role [12–14].

Despite RTW after THR/TKR becoming an increasingly important issue, little is known about what factors impact on the effectiveness of RTW for these employees [15]. In their review of factors influencing employment outcomes for employees undergoing THR/TKR, Malviya et al. [16] concluded that there was a need for qualitative research regarding the RTW of this patient group from an employer perspective. The aim of this study was therefore to explore the experiences of employer stakeholders in supporting employees undergoing THR/TKR. This research was conducted as part of a larger study to design an occupational advice intervention for this group of patients and their employers [17].

## Methods

Ethical approval was obtained from the University of Nottingham Faculty of Medicine and Health Sciences Ethics Committee. Qualitative methodology was used and data collected via semi-structured interviews. Participants were eligible if they had managed employees or staff undergoing THR or TKR within the previous 12 months. As a variety of individuals in the workplace are involved in managing employees undergoing surgery, the terms ‘employer’ or ‘employer representative’ were used when recruiting participants. The sampling strategy included large organisations; small and medium-sized enterprises; the public, private and third sector; and manufacturing and service sectors. It was anticipated that a purposive sample of 24 workplace representatives (WRs), including line managers, Human Resources (HR) representatives and Occupational Health (OH) practitioners, would provide sufficient diversity of views and experiences. The recruitment strategy has been reported in detail elsewhere [18]. However in brief, strategies included approaching previous research participants and those known to members of the research team; emailing organisations from Chambers of Commerce databases; contacting organisations via business networks; ‘cold calling’; and via a study Twitter account. The interview schedule was informed by relevant literature and the experience of the study team. Open questions and prompts were used, with new topics being added as the interviews progressed. A selection of the key topics included in the schedule are provided in Table 1.

**Table 1:** Interview topics with example questions

Topic	Example question
Employer experience	Have you had any training in managing peoples’ health at work?
Management	Could you describe your role in the return to work process for employees who have undergone THR/TKR? Could you give examples of how this was achieved?
Procedures	What are the sort of policies and procedures, including things such as phased returns and sick pay, that are in place to support a return to work?
Role of occupational health	Do you have Occupational Health cover? If so, how does your OH representative assess what activities an employee can or can’t do after a hip or knee replacement?
Employer effecting a return to work	In terms of the advice that you give, and the role that you play, how did you feel about taking on the responsibility of effecting a return to work for the employee?
Work demands	Are there specific work demands that would influence return to work?
Barriers and facilitators to RTW	What things make/could make it difficult for employees who have had knee or hip replacement to return to work? What things make/could make it easier for employees who have had knee or hip replacement to return to work? Why might some employees RTW more effectively than others?

Interviewees were offered a face-to-face or telephone interview at a time convenient to them. Interviews were digitally recorded, transcribed verbatim by an approved provider and checked by the researcher conducting the interview. Data were analysed thematically using the Framework Method [19]. The analysis started mainly deductively from pre-set aims and objectives and conducted within a realist/constructionist paradigm, focusing on semantics rather than identifying themes at a more latent or interpretative level. Codes were developed by considering each line of the transcript to summarise the participants’ views and experiences, then reviewed and refined by the research team. To manage the data systematically, Nvivo 10, a qualitative software package was used to code each transcript. Matrices were then developed using Word to present the data in a case by code format. Potential overarching themes and categories or sub-themes were identified individually by the research team, then agreed collectively through comparison between and within the cases.

## Results

In total, 25 employers and their representatives were recruited and interviewed (participants are henceforth referred to as WRs to encompass their varying roles within their organisations). Interviews took place between September 2016 and June 2017 and were conducted and analysed by three of the authors; CC, MN and FN. Nine interviews were conducted face-to-face and 16 by telephone. The mean length of interview time was 36 min. The demographic profile of interviewees is shown in Table 2.

**Table 2:** Demographic profile of employer participants

ID no.	Relationship to employee	Sector
01	Occupational health physician	Various
02	Occupational health physiotherapist	Manufacturing
03	Human resources	Transport
04	Employee relations	Higher education
05	Colleague	Private health provider
06	Occupational health nurse	Local government
07	Manager	Higher education
08	Human resources	Transport
09	Managing director	Manufacturing
10	Human resources	Service
11	Managing director	Service
12	Manager	Local government
13	Occupational health nurse	Leisure/hospitality
14	Human resources	Leisure/hospitality
15	Manager	Leisure/hospitality
16	Manager	Central government
17	Occupational health advisor	Manufacturing
18	Manager	Manufacturing
19	Manager	Primary education
20	Staff liaison manager	NHS trust
21	Human resources	Retail
22	Manager	Hospitality
23	Manager	NHS trust
24	Human resources manager	NHS trust
25	Human resources	Further education

Two main themes were identified as ‘Accommodating the Employee’ and ‘Barriers and Facilitators to RTW’. Each had a number of subthemes, presented in Table 3.

**Table 3:** Themes and sub-themes identified through analysis of the interview data

Main theme	Sub-theme
Accommodating the employee	Supporting employees prior to surgery
	Modifying hours and duties
	Changing equipment and environments
	Offering alternative roles
	Assistance from other workers
	Accommodations—temporary or permanent?
Barriers and facilitators to return to work	The pros and cons of an occupational health service
	Lack of advice and support from orthopaedic teams
	The limited role of the GP
	Employee motivations and drivers
	The impact of workplace size and structure
	Factors relating to surgery and postoperative care

### Accommodating the Employee

This theme encompasses the ways in which the employer supported and accommodated their employee both pre and post-surgery.

#### Supporting Employees Prior to Surgery

Participants described modifications made to the employee’s role, and the provision of equipment, or assistance with tasks including changes implemented prior to surgery.

…we would put reasonable adjustments in place. So to keep them within the workplace and to keep them in as normal a routine until unfortunately sometimes they’re unfit to carry on work until the surgery takes place – 21 Human Resources

Modifications prior to surgery included the provision of lighter and more manageable work related equipment, and colleague support, in some cases enabling the employee to continue working until the day of surgery. Some employees had had a change in role as they were physically incapable of fulfilling them prior to surgery. Other employees had reportedly managed their work effectively without accommodations up until surgery, with the WR unaware that the employee was experiencing problems carrying out their role, or it was perceived that the employee might be reluctant to disclose this information.

I think he hid it very well…he was a keen tennis player, I think obviously it was becoming an issue with him playing tennis and less of an issue in work. So it was relatively a surprise when he said oh I need to get my hip replaced. OK, so prior to that I hadn’t really been aware of any restrictions – 18 Manager

Others had taken substantial sick leave prior to surgery. Some WRs reported that they might have considered making accommodations for the employee if the GP had advised them accordingly. WRs thought that the GP was more likely to sign the employee off sick until the time of the surgery rather than suggest work modifications that could maintain them in the workplace.

#### Modifying Hours and Duties

Some employees had returned to work by phased return, either in isolation or in conjunction with amended duties. The phased return could be a simple reduction in hours building up gradually over the first few weeks, up to very complex arrangements, incorporating un-used annual leave allocations and public holidays. These arrangements were generally designed with OH if available, but then implemented and reviewed by the manager in conjunction with the employee.

So we had guidelines to be honest from occupational health but we sort of, we adjusted them as we felt fit based on how he felt…. And then we effectively had weekly reviews and increased his activity as he felt appropriate, and also his hours – 18 Manager

Amended duties could include the restriction of heavy lifting; restricting work to one floor level so as to reduce the need to use stairs; the provision of trolleys and light-weight equipment. This was particularly the case with manual jobs such as cleaning, and maintenance roles involving carrying, kneeling and moving heavy equipment. With other occupations involving heavy, factory or warehouse based work, some OH practitioners advocated that there should be no manual work undertaken whilst the employee was undergoing rehabilitation. These employees were given time to re-integrate into their previous role, whereas employees in other organisations remained within the same work environment but only took on work which did not involve certain postures, e.g. kneeling, or particularly physically onerous tasks. Other employers provided respite for employees from the pressure of a mentally demanding workload.

I suppose our job isn’t physical, but it’s very mental…. What my main aim was to wait for him to give us the OK, and then possibly space out his appointment times so that his day wasn’t overfilled or overtaxing on his return…. So I kept – I selected what he got involved with and what he didn’t get involved with if you like… – 5 Colleague

#### Changing Equipment and Environments

WRs referred to providing additional or adapted equipment and furniture to facilitate RTW. Changes included the provision of perching stools allowing employees who would normally stand all day to take seated breaks, or by alterations to desk and office furniture, such as adjustable office chairs, seating wedges and footstools.

I referred her to occupational health for an assessment on her coming back to work and if we needed to put any adjustments in place. We did do a desk risk assessment with her. You know, just to check her chair was OK and the desk and she was sitting right and things like that. And did she need a board, you know, one of those footboards. So, we did all that when she came back – 24 Human Resources

Other WRs described concessions on workplace parking allowing employees to park closer to their place of work, sometimes allowing parking in disabled bays. Some provided taxis or arranged lifts to and from work for the period that employees were unable to access their usual mode of transport.

WRs might move employees to a different work area in order that they could access facilities more easily, or advise the employee to use lifts rather than stairs to access their workspace. Some employees were relocated to a different work site nearer to their home until they had recovered to a point that they could travel independently. Others were given the opportunity to work from home initially, if their role made this possible.

#### Offering Alternative Roles

It was reported that some employees requested to return to a different job as they did not feel able to satisfy the requirements of their previous role. WRs might be willing to concede in order to facilitate a timely RTW. Additional training might also be arranged.

Other employers opted to move the employee into another team with a less physically demanding role. This included support from work colleagues,

She’s been having a phased return back to work…we’ll give her two extra afternoons now from next week…being a primary school, because obviously they teach all lessons and her class is being covered for PE lessons probably for the next six months just to make sure there’s no running about on her part – 19 Manager

Smaller businesses found it more difficult to offer alternative roles, and resorted to what they perceived to be ‘light duties’ such as cleaning, sweeping and delivery driving to enable the employee to return.

#### Assistance from Other Workers

Although there was an expectation that colleagues would provide informal support for those returning to work following surgery, some WRs facilitated this by requesting assistance from other staff.

We’ve got staff who will help her take, when she’s finished her day or when she’s beginning her day, they will help her take stuff to and from her car. She does have one of those wheelie trolleys but even so pulling it isn’t easy, so we support her as a team – 20 Staff Liaison Manager

Colleagues might be asked to step in and assist with certain activities, with one employee returning to work initially on a supernumerary basis so that she was effectively an extra ‘pair of hands’.

#### Accommodations: Temporary or Permanent?

Some participants reported that the employee had not returned to work, or was back at work but remained on modified duties.

So that’s how we’re accommodating things at the moment…. I suppose if it is something that’s going to be permanent and she’s not going to improve any further, then maybe we may need to look at some equipment to help her do her job, you know. Would she need a special chair for sitting in a delivery room, you know, things like that, and I would look to [OH provider] to help me with that – 23 Ward Manager

Issues around the inability to kneel post-surgery resulted in employees having to be relieved of some of their previous duties. This was particularly the case in physically demanding roles. Not all employees were able to RTW even with work adjustments or altered hours as the problems they were having were insurmountable at that point in their recovery.

…she’s been off about three months so with that altogether it took a lot longer for her to come back into it from that point of view. We are quite quick at getting them back in actually because, a lot of the time they do, as I say, come on this phased return bit so they don’t actually do the full role. But she was just not fit even for amended duties. – 13 OH Nurse

In some cases employees had returned to work but were unable to fulfil their duties and as a consequence had gone back on sick leave.

### Barriers and Facilitators to Return to Work

This theme incorporates the strategies and processes which facilitated RTW following THR/TKR and highlights those aspects which were seen to impede a timely return to the workplace.

#### The Pros and Cons of an Occupational Health Service

There was a view that organisations with an on-site OH service were at an advantage in supporting RTW due to their perceived better understanding of the job demands. OH advisers could also give reassurance to employers that they were acting in accordance with best practice, and complying with current employment legislation.

…occupational health are there to support and guide, and I think that’s really important when we’re talking about people who are working outside in the community or in a ward environment. I think it’s important for all people, but more so people who are patient-facing – 20 Staff Liaison manager

Some organisations reported having regular clinics held with an OH/company doctor rather than an on-site service. In some organisations all employees undergoing THR/TKR would be referred to OH. However, in other cases referral was at the discretion of the manager, and not necessarily before the employee had returned to work.

OH input was generally positively received, however not all interviewees valued every OH intervention but felt it necessary if they had received insufficient medical advice from the employee’s GP or surgeon, or as a ‘back up’.

I have no idea what we pay for the service through our occupational health provider, but to me I’m perfectly capable of reading that on the intranet myself, so has it added value having it in a headed letter from a health provider, probably not…. So has it helped me manage that absence better through the occupational information? Probably not, but it gives me the reassurance that should I need anything I’ve got access to it… – 16 Manager

Some questioned whether OH practitioners had sufficient in-depth knowledge of the employees’ work tasks, that employees themselves might have a negative perception, or misunderstand the role of OH, limiting their potential cooperation. Those interviewees who represented OH often felt that their departments were under-resourced, however there was a perception that, as medical staff such as surgeons and GPs were not sufficiently trained in work-related issues, OH services were imperative to a successful RTW.

#### Lack of Advice and Support from the Orthopaedic Team

There was a consensus amongst WRs that there was insufficient communication between the orthopaedic team and themselves. It was reported that they would value the opportunity to discuss the specifics of their employees’ recovery from surgery, the anticipated longevity of the new joint, the time scales involved and what the employee could and could not do on RTW.

I would like them to be able to say whether he could drive, whether he could walk, whether he’d be in – how much pain they would expect him to be in. I think it would be more just the day to day things and then we could get occupational health to do an assessment related to his particular job – 25 Head of HR

They were also unclear as to what the final expected functional outcome might be, with some employers expecting their employee to be ‘fixed’ and working more productively than they were pre-surgery.

WRs felt too reliant on employee-reporting of their recovery leading to suspicions that employees might be imparting misleading or misguided information, or even manipulating the situation to fulfil a personal agenda. They felt that, once the decision to operate had been made, that the surgeon should advise the employee and their employer on how the procedure might impact on work. As there was usually at least a two month gap between the surgical decision and the operation, this would give the employer time to plan ahead to provide staff cover, and prepare for any necessary modifications.

Yeah I think once the person has been informed by their GP that the recommendation is a knee or hip replacement and that they’ve agreed to it, therefore it’s definitely going to happen, at that point because it’ll be a few months before it actually happens, but at that point there should be some understanding for the manager and the person themselves and what this means in terms of work – 12 Commissioning Manager

However, other WRs felt that they would prefer to receive this advice post-surgery when the result of the operation and any potential complications were known as this would give the employer a more accurate prediction of the RTW date.

#### The Limited Role of the GP

Interviewees reported that GPs were variable in the RTW support they provided, that GPs were limited in time and expertise, and reliant on the patient for information about work issues. Although fit notes were perceived by some employers to be of benefit, others disagreed and felt that the information provided on fit notes was often limited. Employers felt that the fit note provided the opportunity for GPs to make recommendations on possible work modifications but these were seldom made.

There was also a view that GPs were inclined to be overcautious, or might raise an employee’s expectations inappropriately, or only consider the employee’s current job, rather than any potential alternatives. Some employees were thought to consider the fit note as ‘gospel’, rather than advisory, although likewise the employer might also be reluctant to act against fit note advice,

And if the, sometimes the GP will put down a long time on a fit note, and that person takes that fit note as god. They won’t veer from that: my GP says I can only return back to work after that date. And you cannot make them change their minds about coming back to work any sooner. Actually you can come back to work before that date, because you can do X Y and Z and you just show me you can do X Y and Z – 6 OH nurse

As a result WRs were more likely to rely on OH advice if available, or would request further information from the GP. However, obtaining more detailed advice from GPs was limited by communication systems, often making the process protracted and problematic.

Some WRs felt that they needed to get GP approval for a RTW, others did not necessarily pay too much attention to the fit note.

And occasionally you sort of, we’ve gone and said to individuals, individuals who are going to come back to work against the GP’s advice, which is interesting, or come back early because I feel great. So the doctor’s signed me up for a month but after three weeks I feel great so I’m going to come back in and that creates issues at work when you’ve got people working against medical advice – 18 Manager

A number of WRs expressed concern regarding the pain relief medication prescribed by GPs to employees following surgery, particularly if this presented a risk to the employee or others due to the possible effects of the medication on their ability to perform their role. This was particularly the case with roles involving public safety or the use of heavy machinery. It was felt that this information should be imparted to the employer as the patient was not always aware of the effects of the medication that they were taking.

#### Employee Motivations and Drivers

The employee’s personal characteristics were perceived to be potentially both a hindrance and a help to their effective RTW. Some employees were keen to RTW as soon as possible—in some cases too early—due to the loss of their usual routine, and boredom, finding it difficult to adapt to not being at work,

So it is hard telling somebody that they’ve got to be off that little bit longer when they’re desperately wanting to return. It is difficult, especially when they’re pleading to you and they’re wanting to come in – 13 OH nurse

Others were keen to return due to job demands and responsibilities, or for reasons of finance or job security.

WRs recognised that it was important to re-establish a work routine as early as possible, and that some employees might be anxious about returning to work. Other employees were reported to have delayed surgery because of anxiety about the operation itself.

WRs thought that those in manual jobs might struggle to consider moving onto lighter, more sedentary office-based duties, or be reluctant to return to jobs which they felt might have contributed to their arthritis.

Employees’ compliance with rehabilitation and self-management of their health was seen as a key factor in RTW. There was a view that some employees needed more active support in the recovery process, particularly those people who do not have anyone to support them at home or are lacking in motivation and enthusiasm to RTW.

Proximity to retirement was also considered a factor, and concerns that RTW might impact on the new joint.

Because they’ve only either got a few months left or a couple of years left, and they just think do you know what, it’s not worth coming back and heaven forbid but doing any more damage – 3 Human Resources

Employee beliefs about the RTW process were thought to create obstacles, for example not knowing or understanding about phased returns, or any of the other work modifications and accommodations that might facilitate the process. However the motivation of the employee to RTW was seen to be paramount.

#### Impact of Workplace Size and Structure

Some interviewees took the view that the size of the organisation had an impact on the employee’s RTW, for example, managers in smaller organisations might be less skilled in the process. Managers might also have little access to support systems—and less experience of joint replacement surgery.

I guess in a bigger business you’d have a HR department or you’d have a HR person who is allocated to each member of staff or whatever…. No, I think it’s probably, given my limited experience on it because we’re a small business and we haven’t been through it other than this one recent case – 11 Managing Director

However even the representatives from larger organisations felt that line managers might not be aware of the organisational support available to them in managing RTW. Those larger organisations that had their own on-site rehabilitation service were perceived to enable line managers to better manage an optimal RTW experience, and provide ongoing rehabilitation in the workplace. However, in smaller businesses, there were seen to be fewer options for work adjustments and changes to how work was organised.

Very large organisations were more likely to have set procedures for employees returning to work following THR/TKR as they had more experience of the issues involved. It was observed that lengthy periods of sickness absence were more easily accommodated by larger organisations as they had more capacity to cover periods of sickness absence.

…can’t remember how many months it was now, but it was definitely three to four months if not longer where she worked in this other team before planning her return into her post – 20 Staff Liaison Manager

Employees in smaller organisations or teams were thought to be less comfortable about taking sick leave because of the demands that their absence would place on their colleagues and employer. However there was a perception that some organisations might be less supportive than others, and that some posts were more difficult to provide cover for. Even within the same organisations, sick pay arrangements, phased returns or access to health schemes might differ between departments and the seniority of staff, which consequently impact on the success of the RTW.

Office-based and non-manual work roles were seen as easier to return to. However some interviewees perceived that adjustments might also be required for office-based work particularly in terms of work station assessments and altered working practices to ensure workers remained mobile.

Once an individual had returned to the workplace it was thought that colleagues might need prompting to be supportive with any adjustments and accommodations, and even employees in sedentary tasks still needed to be able to travel to work and be mobile within the workplace.

#### Factors Relating to Surgery and Postoperative Care

Factors relating to surgery itself were perceived to impact on RTW. Where there had been complications such as infections or blood clots, or ongoing symptoms and after-effects of surgery such as stiffness, pain, swelling, low mood and fatigue, a full and successful RTW was protracted. However some WRs were surprised as to how well their employee was coped considering the amount of physical effort required to fulfil their role.

The impact of successive joint replacements on sick leave was also a consideration, and perceptions of insufficient or delayed post-operative care and physiotherapy, with some employers opting to take advantage of the services of private providers associated with their organisation.

they’re waiting three weeks for physio-that’s three weeks of their time lost here…he wasn’t referred for physio. He was just given exercises. Whereas everybody else I’ve known has been referred to a physio…. So I think unfortunately it’s a postcode lottery is the impression I get, but I could be wrong. And we’ve actually had to say to this guy well look, we can get you physio private…you can claim up to six sessions back – 3 Human Resources

Surgery undertaken privately was seen by some to facilitate the process, and that NHS delays and cancellations were a hindrance, however, others had not experienced any such problems.

For large organisations with highly structured RTW policies for THR and TKR, the variation in expected duration of sickness absence and RTW advice between different surgeons and Trusts was seen as a potential hindrance.

And I suppose the difference then in NHS is that you may then have some people that are off for six weeks, some people are off for eight weeks, maybe ten weeks and it could potentially then cause problems – 2 OH Physiotherapist

## Discussion

This is the first study to report on the experiences of workplace representatives in managing employees undergoing THR/TKR. In contrast to previous findings [20] which reported that employers make RTW decisions in isolation, our study suggests that the reverse is true for WRs effecting a RTW after THR/TKR. They describe RTW planning which relies heavily on the needs and wants of the employee. The RTW plan was generally devised jointly between the employer, employee and OH practitioner, if available. As with the findings of Stahl et al. [21], OH was reported to play a significant role in the RTW process through the undertaking of workplace assessments and provision of advice on possible accommodations to be made. In the absence of any input from the GP or other medical practitioner, WRs found this support invaluable, however, in our study line managers were often responsible for individualising this advice for the employee. Assistance reported to be available to employees ranged from colleague and co-worker support only, to onsite OH and rehabilitation facilities. Despite the disparity in WR experience and the support available to them, the majority still reported that RTW was hampered by the absence of guidance or support. WRs therefore reported having to rely on ‘gut instinct’ and surmise when attempting to facilitate a timely and safe RTW.

Although employers interviewed in our study alluded to their consideration of business pressures, they reported that their priority remained with the employee and the facilitation of a RTW which fulfilled both the needs of the employee and the employer. Despite previous research [22] suggesting that employers perceived the RTW process as burdensome, WRs in this study, despite their concerns about lack of information from medical practitioners, were positive and motivated to get their employee back into work at the earliest opportunity but within the confines of what was acceptable to the employee themselves. However, we acknowledge our sample may have been biased in that interviewees may have wanted to display a positive image, and that WRs who were less supportive of employees, or concerned about scrutiny, may have been less likely to participate.

Our study recruited WRs across a range of organisations, both public and private, including those employing as few as 10 people up to those employing several 1000. We believe that study credibility was increased by recruiting participants with various roles and experiences even though this diversity made it unlikely that we reached data saturation. Individual interviews encouraged participants to share their individual experiences and perceptions without being influenced by the presence and views of other participants which might have arisen in a focus group setting. Use of the Framework Method provided a straightforward and explicit approach to thematic analysis, facilitating transparency. Transferability was facilitated by providing detailed description of the method of selection; the process of analysis; the characteristics of the participants and the inclusion of quotations. None of the researchers had conducted research with this client group before, although they had conducted qualitative studies with employers previously which may have both informed and influenced their approach to questioning and analysis. In addition, a more latent approach may have yielded different findings and interpretations. Dependability was increased by having the same three researchers conducting, checking and analysing the data. One potential weakness of the study could be that the themes identified were not confirmed by the participants but unfortunately this was impractical within the confines of the wider study due to time, cost and ethical constraints. For the purpose of this study, no distinction was made between employees undergoing hip or knee replacement. Evidence suggests that RTW after TKR is less successful than for THR, and that strategies to effect change should consider both types of surgery separately [23], thus future research is indicated to explore RTW experiences according to the joint being replaced.

In the UK there is no legal requirement for employers to provide OH services. However health and safety legislation places a duty on the employer regarding the health, safety and welfare at work of their employees. As a consequence, most public sector employers provide an OH service but overall only a small proportion of the UK working population have access to OH. To address this limitation a government-funded ‘Fit for Work’ initiative was implemented to offer free occupational advice for employers and GPs in supporting employees in RTW. However, an evaluation of this service reported limited uptake [24], and the government has recently acknowledged that the current model of OH provision is not meeting the needs of employers and individuals [25]. Despite surgeons and GPs being able to advise employers on work modifications via the ‘fit note’, in our study, WRs reported that this rarely happened, and it seemed that the fit note was not used as intended by either employees or employers. This resulted in an over reliance on employee reporting of their recovery and potential work restrictions. Limitations in the use of the fit note by GPs and hospital doctors has been reported elsewhere [26, 27]. The UK government has recently committed to reforming the fit note in order for it to be used more effectively [25]; further studies are indicated to explore the impact these reforms might have on fit notes provided to patients undergoing elective surgery.

In our study, the main focus was on employees undergoing arthroplasty. Employees had not necessarily returned to work at all following surgery, whilst some were making slow progress, and there was some uncertainty amongst participants as to what level of work ability could be expected. In some cases, complications of surgery delayed recovery. There has been little research to support the effectiveness of joint replacement surgery in maintaining and/or increasing work ability in comparison to nonsurgical approaches for those with OA. Research by Skou et al. [28] suggests that patient education and training can be effective as an adjunct or even an alternative to surgery, and that although surgery may result in greater pain relief and functional outcomes, there is also an increased likelihood of adverse events. However, their study did not focus on RTW specifically.

Patients who undergo THR and TKR accrue substantial sick leave both before and after surgery [29]. Accessible workplaces are a predictor of RTW following THR and TKR [30]. In our study, some participants reported how they had been able to make modifications to enable employees to remain in work before surgery, thus reducing preoperative sick leave, which is another reported determinant of RTW in THR and THR [23]. However, participants also indicated that some employees might be reluctant to disclose their health problem thus limiting the opportunity to access help. Gignac et al. [31] have highlighted the barriers to disclosure of arthritis at work, and other studies have suggested that some employees may decide not to proceed with surgery because of work factors [32]. This is of considerable concern as early interventions and good communication might delay or avoid the need for surgery [33]. Furthermore, in contrast with traumatic health conditions, elective surgical procedures such as THR and TKR would seem to present employers, employees and clinicians with an ideal opportunity to make RTW plans in advance. When comparing the work outcomes in THR and TKR, research suggests that there are differences in time to RTW, and that a predicted duration of absence post-surgery and expected level of recovery would be invaluable in the RTW planning process for both employers and employees [34, 35].

The Royal College of Surgeons publish online guidance for patients recovering from surgical procedures including THR or TKR [36], which incorporates advice on RTW, but this guidance is not directed at, and unlikely to be accessed by, employers. It also cannot advise employers on the needs of the individual. It would therefore appear that clinicians need to consider RTW outcomes for their patients, how these can be optimised with further research being undertaken to explore how this might best be achieved.

## Conclusion

WRs are motivated to effect a supported and timely RTW for employees undergoing THR/TKR but feel they receive insufficient advice and support on how to achieve this. Despite the existence of national guidance, information is not reaching the intended recipients, and may not meet the needs of the individual employee. Workplace strategies are required to signpost employers to existing sources of guidance and support, and to facilitate communication with the medical practitioners responsible for the employee’s recovery. Without these measures we will not improve the RTW experience of those undergoing THR/TKR. This would represent a missed opportunity for the growing numbers of employers and their employees.
